# Southern Medical College

**Published:** 1879-02-20

**Authors:** 


### *SOUTHERN MEDICAL COLLEGE.*

The Board of Trustees of the Southern Medical College met in Atlanta on the 12th inst., and was duly organized by electing as officers of the Board T. S. Powell, M.D., President; Rev. J. J. Toon. Vice-President; R. C. Word, M.D., Secretary; and Judge S. B. Hoyt, Treasurer.

A Constitution and By-Laws were adopted; a Finance and Building Committee appointed, and the Treasurer directed to open books for the procurement of stock. The Building and Finance Committee was instructed to select suitable ground and estimate the cost of a proper building, and report to the Board as early as possible. The Treasurer was authorized, by himself and his appointed Agents, to solicit donations for the Institution.

All correspondence in relation to this Institution may be addressed to the Secretary of the Board.

